# Evaluating 3D-printed bioseparation structures using multi-length scale tomography

**DOI:** 10.1007/s00216-023-04866-6

**Published:** 2023-07-31

**Authors:** Thomas F. Johnson, Mariachiara Conti, Francesco Iacoviello, Paul R. Shearing, James Pullen, Simone Dimartino, Daniel G. Bracewell

**Affiliations:** 1 Department of Biochemical Engineering, University College London, Bernard Katz, London, WC1E 6BT UK; 2 Institute for Bioengineering, School of Engineering, University of Edinburgh, Edinburgh, EH9 3JL UK; 3 Electrochemical Innovation Laboratory, Department of Chemical Engineering, University College London, Torrington Place, London, WC1E 7JE UK; 4 Fujifilm Diosynth Technologies, Belasis Avenue, Billingham, TS23 1LH UK

**Keywords:** X-ray imaging, 3D printing, Gyroid, Flow simulation, Tortuosity

## Abstract

X-ray computed tomography was applied in imaging 3D-printed gyroids used for bioseparation in order to visualize and characterize structures from the entire geometry down to individual nanopores. Methacrylate prints were fabricated with feature sizes of 500 µm, 300 µm, and 200 µm, with the material phase exhibiting a porous substructure in all cases. Two X-ray scanners achieved pixel sizes from 5 µm to 16 nm to produce digital representations of samples across multiple length scales as the basis for geometric analysis and flow simulation. At the gyroid scale, imaged samples were visually compared to the original computed-aided designs to analyze printing fidelity across all feature sizes. An individual 500 µm feature, part of the overall gyroid structure, was compared and overlaid between design and imaged volumes, identifying individual printed layers. Internal subvolumes of all feature sizes were segmented into material and void phases for permeable flow analysis. Small pieces of 3D-printed material were optimized for nanotomographic imaging at a pixel size of 63 nm, with all three gyroid samples exhibiting similar geometric characteristics when measured. An average porosity of 45% was obtained that was within the expected design range, and a tortuosity factor of 2.52 was measured. Applying a voidage network map enabled the size, location, and connectivity of pores to be identified, obtaining an average pore size of 793 nm. Using Avizo XLAB at a bulk diffusivity of 7.00 × 10^−11^ m^2^s^−1^ resulted in a simulated material diffusivity of 2.17 × 10^−11^ m^2^s^−1^ ± 0.16 × 10^−11^ m^2^s^−1^.

**Graphical abstract:**

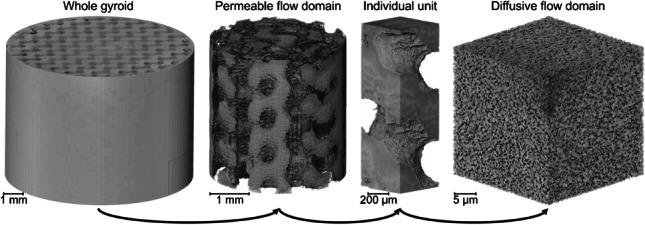

## Introduction

Medicine manufacturing is a complex and expensive process at scale, requiring multiple unit operations to produce and purify a product of interest to required standards. Recent advances in emergent modalities, for example, lipid-enveloped particles, present major therapeutic advantages but also challenges during bioprocessing when compared to conventional products such as monoclonal antibodies [1, 2]. Also, the onset of the “omics” requires new stationary phases with improved peak capacity able to fractionate such complex samples [3, 4]. In order to maximize bioprocess efficiency, effective unit operations must be selected and optimized, often using a chain of the most suitable filters and chromatography columns for purification [5, 6].

Chromatographic unit operations rely on chemical and physical characteristics to perform bioseparations [7, 8]. Structure relates directly to function and performance, which for chromatography columns, spans a hierarchical structure consisting of (i) the space within the packed bed and (ii) within individual beads. Both length scales exhibit variability and often lack definitive control over physical characteristics.

Additive manufacturing provides an approach whereby the geometry of a material can be precisely designed and fabricated as specified by CAD models [9–12]. This enables physical characteristics such as channel size and morphology to be designed that meet the intended function and are optimized for performance [13–15]. The ability to 3D-print porous materials through polymerization-induced phase-separation enables the formation of a porous network at the nanoscale, essential to increase the surface area to volume ratio for the adsorption of biomolecules [16].

Additive manufacturing of porous materials enables the fabrication of hierarchically porous media, with control over the microscale morphology through CAD design, and control of the nanoscale through chemical composition. This enables the intelligent design of novel structures that can be specifically tailored to the product of interest and rapidly prototyped, including chemical and physical properties that influence diffusive and permeable flow to improve bioseparations performance. By 3D printing structures for bioprocessing, several physical limitations of existing chromatographic structures may be reduced or eliminated, for example, wall effects and particle size distributions within packed beds. 3D printing materials for bioprocessing has been demonstrated in multiple studies [17–20]. This has included 3D printing ion exchange monoliths not requiring functionalization to bind model proteins, in turn allowing product needs to adapt purification material specifications rather than conventional means, such as dictating 3D-print volume to the process instead of using generically sized columns [17].

Computational fluid dynamics has been applied in examining flow behavior through repeating gyroid structures [21, 22]. This has included a study that focuses on the chromatographic performance and capabilities of repeating geometries as comparative media to conventional packed bed resins [23]. The authors commented on a key advantage being the ability to precisely specify a desired porosity at a scale analogous to the space between chromatography beads. Another study has applied magnetic resonance imaging in conjunction with computational fluid simulations to further compare flow properties between repeating structures to randomly packed beds [24].

High-resolution X-ray imaging techniques have become increasingly capable and popular for viewing and analyzing internal geometries for a wide range of materials. X-ray computed tomography (CT) has been successfully used to enhance the design of battery materials, whereby physical characteristics including porosity and pore size can be evaluated before, during, and after use [25–27].

Bioprocess media including chromatography resins and nanospun structures for adherent cell growth have previously been imaged using X-ray CT at micro- and nanoresolutions [28–31]. Here we combine the capabilities of 3D-printed hierarchically porous media with that of X-ray CT to control and assess the resulting morphology across multiple length scales.

## Materials and methods

### Design and fabrication

Computer-aided design (CAD) was applied to design Schoen gyroid structures using Autodesk (USA) Fusion 360, generating Standard Tessellation Language (STL) files. A Solflex 350 Digital Light Processing Printer (W2P Engineering, Austria) was used for the fabrication of each methacrylate-based gyroid (with 50% designed bed porosity) at specified feature sizes of 500 µm, 300 µm, and 200 µm. All parts were printed with 50 µm layer thickness. Porogens were removed post-printing to create a nanoscale porous substructure [32, 33].

### X-ray imaging

X-ray CT was performed using two scanners, both accessed at the UCL Electrochemical Innovation Laboratory. To image-dried gyroid samples, a ZEISS Xradia Versa 620 (Pleasanton, USA) with a tungsten target was used, achieving a pixel size of 5 µm for whole sample scans and 2 µm when performing interior tomography [34]. A primary accelerating voltage of 40 keV and a power rating of 3 W over 2401 projections were acquired for each dried sample at a 14 s exposure time, with each scan taking 12 h.

**Fig. 1:**
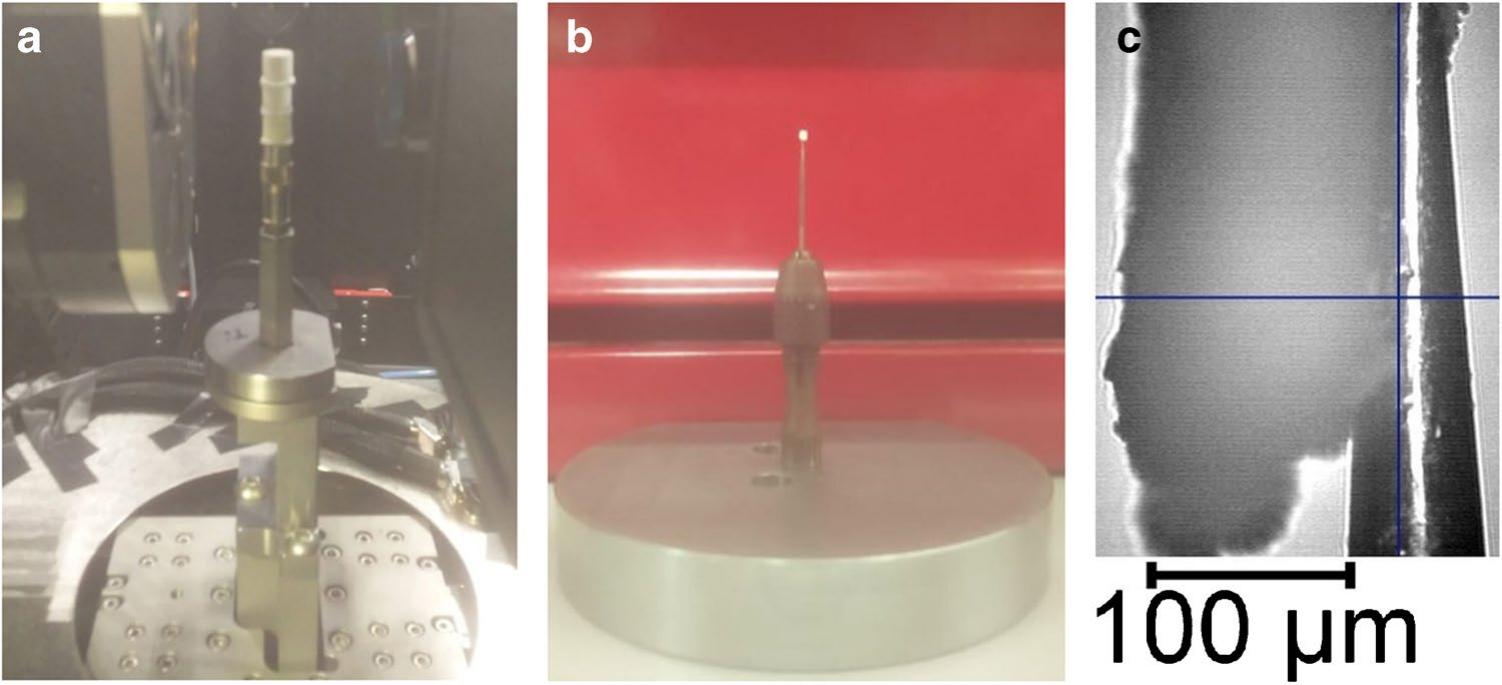
X-ray CT imaging setup. **a** Three mounted gyroids before whole-body scanning. **b** 3D-printed material cut-out adhered to the top of a pinhead and sample holder. **c** Magnified image of the interface between sample (left) and sample-holding pinhead (right)

When visualizing the internal porosity inside of the 3D-printed sample, a ZEISS Xradia Ultra 810 with a chromium target was used at a fixed accelerating voltage of 35 keV [35, 36]. A piece of printed material was cut to dimensions of 1 mm and adhered atop a pinhead for imaging, as displayed in Fig. 1b. A pixel size of 63 nm was achieved for “Large Field Of View” mode and 16 nm using “High Resolution” mode. A 50 s exposure time was applied to acquire 1601 projections per sample, with each scan taking 24 h.

### Digital reconstruction and analysis

Reconstruction of 2D radiographs was performed using ZEISS XM Reconstructor software to produce TXM files. These 3D files were loaded into Thermo Fisher (MA, USA) Avizo® Fire 2022 for processing and analysis [37, 38]. Files were cropped to the appropriate size before applying “non-local means” and “unsharp masking” commands to reduce and remove noise from each volume. The “Interactive Thresholding” command was used to segment material and void phases for analysis before applying further analysis commands, for example, “Volume Fraction” to measure porosity [39, 40].

“Chamfer Distance Map” and “Separate Objects” were used to produce a Pore Network Map that enabled the identification of pore sizes and connectivity [41, 42]. The XLAB plugin was used for “Permeability Simulation Experiment” to visualize and characterize flow through each digital geometry [43, 44]. 3D TIFF files were exported to the MATLAB plugin TauFactor to evaluate the tortuosity factor [45–47].

## Results and discussion

### Comparing design to imaged volumes

**Fig. 2:**
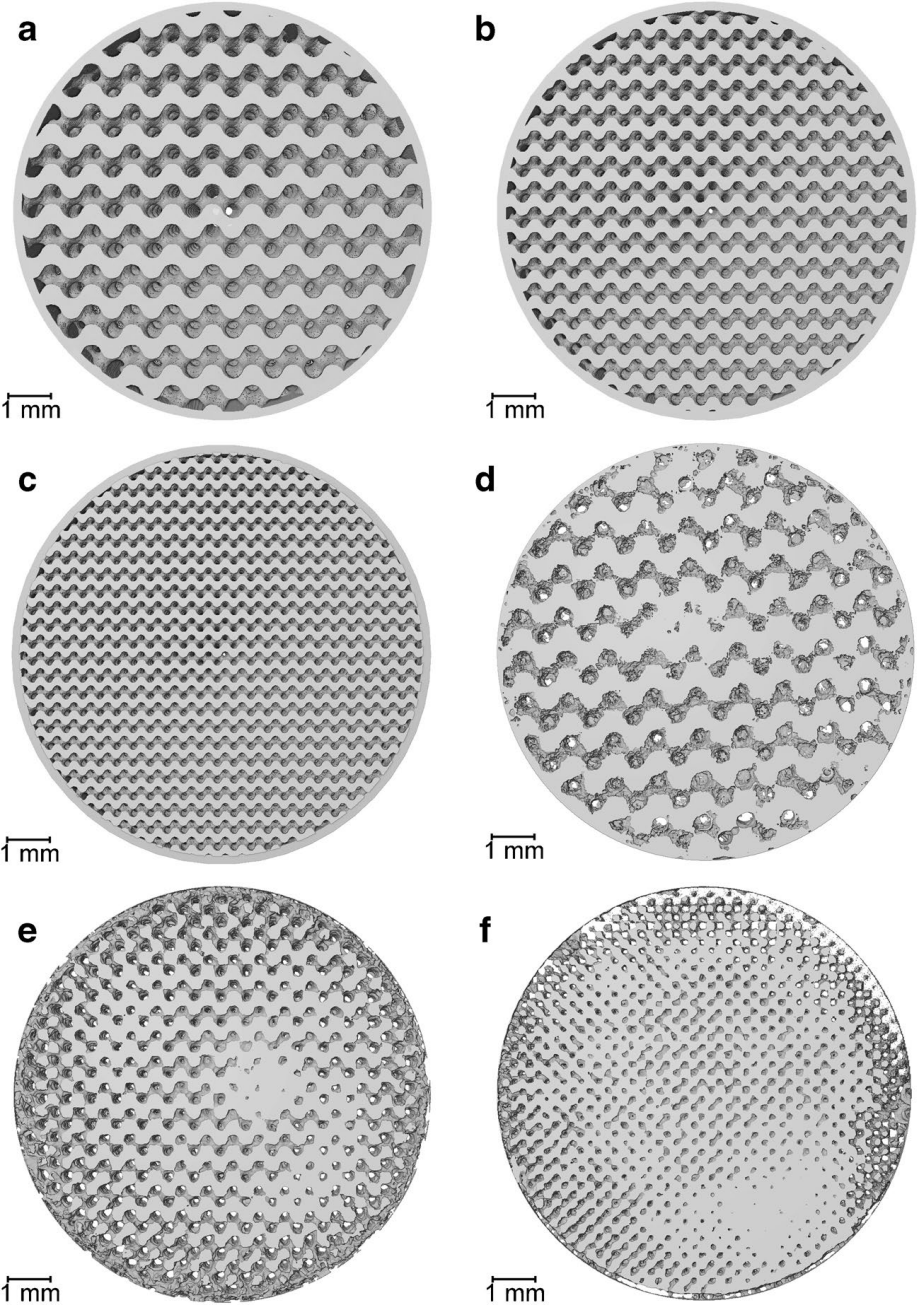
Whole gyroid design and imaging at a 5 µm pixel size. **a** Overhead CAD view with 500 µm feature sizes. **b** Overhead CAD view with 300 µm feature sizes. **c** Overhead CAD view with 200 µm feature sizes. **d** Overhead X-ray CT view of 3D-printed gyroid with 500 µm feature sizes. **e** Overhead X-ray CT view of 3D-printed gyroid with 300 µm feature sizes. **f** overhead X-ray CT view of 3D-printed gyroid with 200 µm feature sizes

X-ray CT was deemed as the most appropriate 3D imaging technique for visualizing whole gyroids due to the high resolutions achievable without requiring sample embedding or destructive sectioning. Figure 1a displays three printed gyroids atop a sample holder in preparation for sequential scanning. Each gyroid design uses identical structurally repeating units, which results in a bed porosity of 50% and provides mechanical strength in addition to providing uniform flow paths both axially and radially [33]. Three gyroid designs with specified feature sizes of 500 µm, 300 µm, and 200 µm were designed and 3D-printed for comparison. CAD renders available in Fig. 2a–c display the visual comparison between designs, where both the material and void phases are equally scaled in size.

Conventional bioseparation structures at a comparative length scale such as packed bed chromatography columns exhibit heterogeneity due to non-uniform particle sizes and wall effects, which have been visualized and characterized in a previous study [30]. By specifying a regular feature shape and size that can then be accurately fabricated through 3D printing, more uniform structural characteristics are theoretically achievable. By X-ray CT imaging 3D-printed materials, direct comparisons can be made to the original designs, with a 3D reconstruction of each feature size available in Fig. 2d–f.

The imaged gyroids in each case display the feature sizes as specified from CAD files throughout the 3D geometry. The capability to design and fabricate intricate structures with confidence enables physical characteristics to be specifically tailored to the needs of the bioprocess product to be purified. Figure 2d–f do display some artifacts that suggest that fabrication presents some fidelity challenges, particularly as feature size moves closer to the achievable 3D printing resolution. Some over-curing of the DLP resin was observed that results in a reduced porous channel diameter, especially in the internal regions of the 3D printed model.

Imaging complex geometries at the microscale can result in reduced signal-to-noise ratios closer to the center of each sample. Printing smaller samples may reduce this concern whilst improving pixel size from 5 µm achieved here; however, producing gyroids with smaller diameters was found to exhibit structural integrity issues during the drying process required for effective X-ray CT acquisition.

**Fig. 3:**
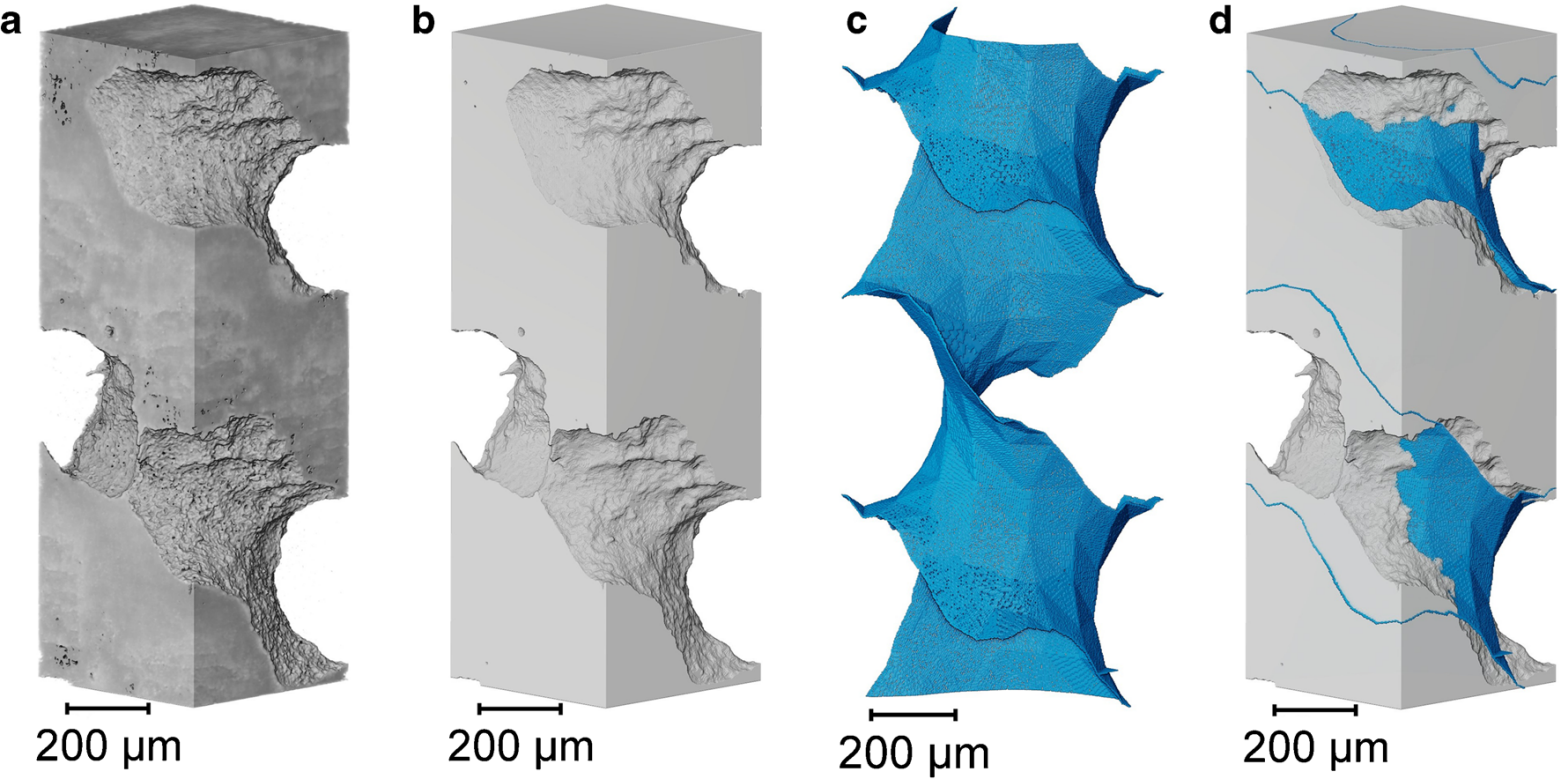
Individual 500 µm unit comparison between designed and imaged volumes. **a** 3D render of internal unit imaged at a 2 µm pixel size. **b** Segmented material phase of imaged unit. **c** CAD design of an individual unit. **d** Design and imaged overlay

In order to examine 3D printing fidelity in detail, internal tomography was performed on a 500 µm feature at an improved pixel size of 2 µm. By focusing on a single unit within the overall gyroid structure, more characteristics such as surface roughness could be visualized. As can be seen in the material phase of Fig. 3a, horizontal striations 50 µm apart are visible, with these being the interface between printed layers. Material and voidage phases are binarised in Fig. 3b.

The imaged dataset was aligned in the same position as the original design file. Figure 3c displays a 3D render of the solid–fluid interface and Fig. 3d overlaying the design and imaged material. Reductions to the designed feature size may become more problematic at the individual unit scale, comparable to an individual chromatography bead within a packed bed. However, it may be favorable to increase the surface area available for fluid to interface with printed material whilst maintaining an ordered flow pattern. Individual features may get closer in size to conventional chromatography beads at improved printing resolutions, which will enable direct comparisons between the two approaches at this length scale.

### Permeable flow analysis

**Fig. 4:**
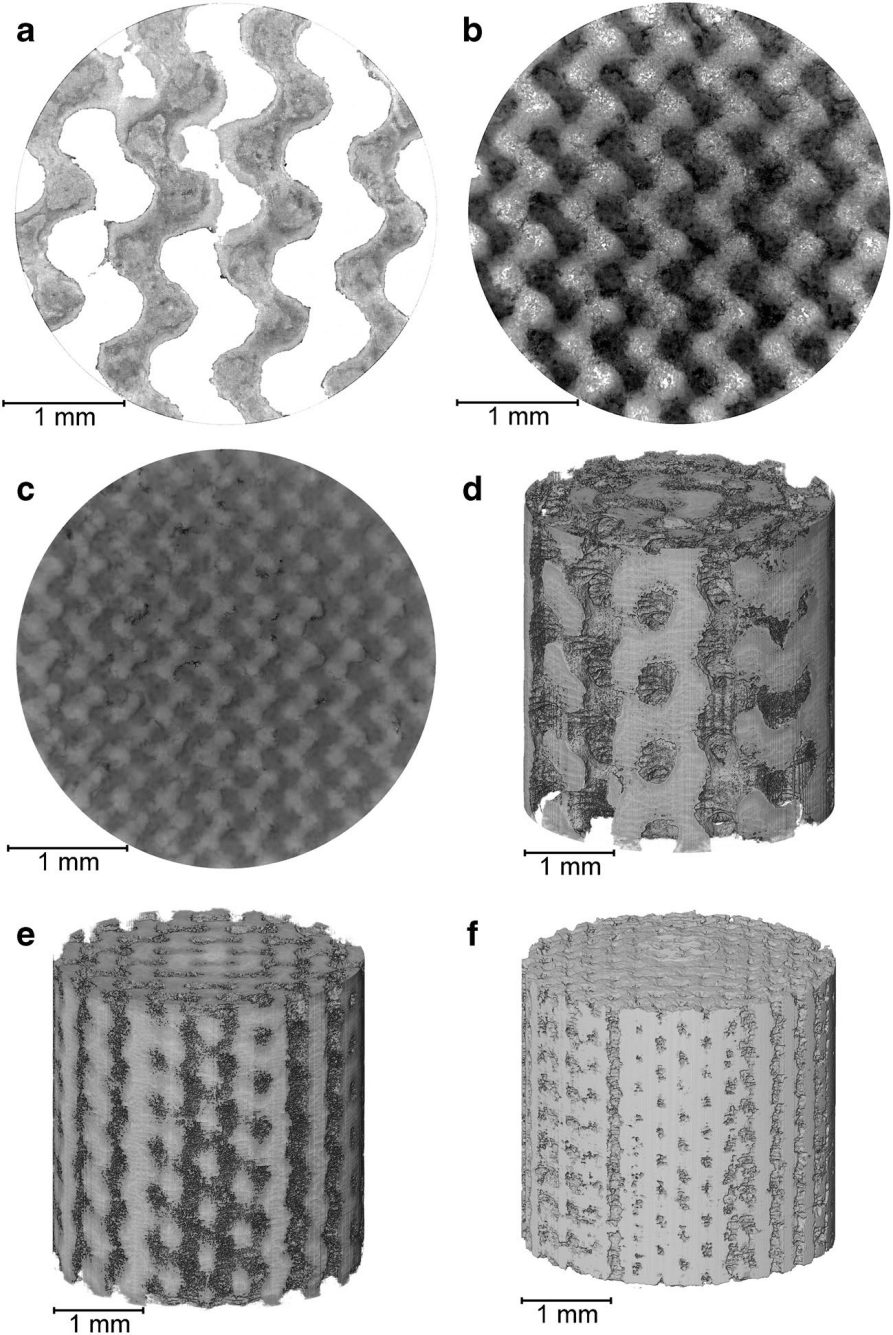
Internal imaging at a 2 µm feature size. **a** 500 µm feature size slice. **b** 300 µm feature size slice. **c** 200 µm feature size slice. **d** 500 µm feature size render. **e** 300 µm feature size render. **f** 200 µm feature size render

3D printing using the gyroid configuration enables complex yet controlled flow paths to be generated within the internal structure; this was imaged at a 2 µm pixel size for all three designs. Imaging at improved resolutions does compromise the field of view and so an appropriate trade-off was required whereby internal features could be identified without imaging an unrepresentatively small volume. Figure 4 displays 2D horizontal slices and 3D renders of each internal subvolume, where again the structural differences can be observed in terms of material and void thickness. In a similar manner to Fig. 3a, the horizontal printing layers are visible in these imaged volumes, particularly noticeable in Fig. 4e–f.

**Fig. 5:**
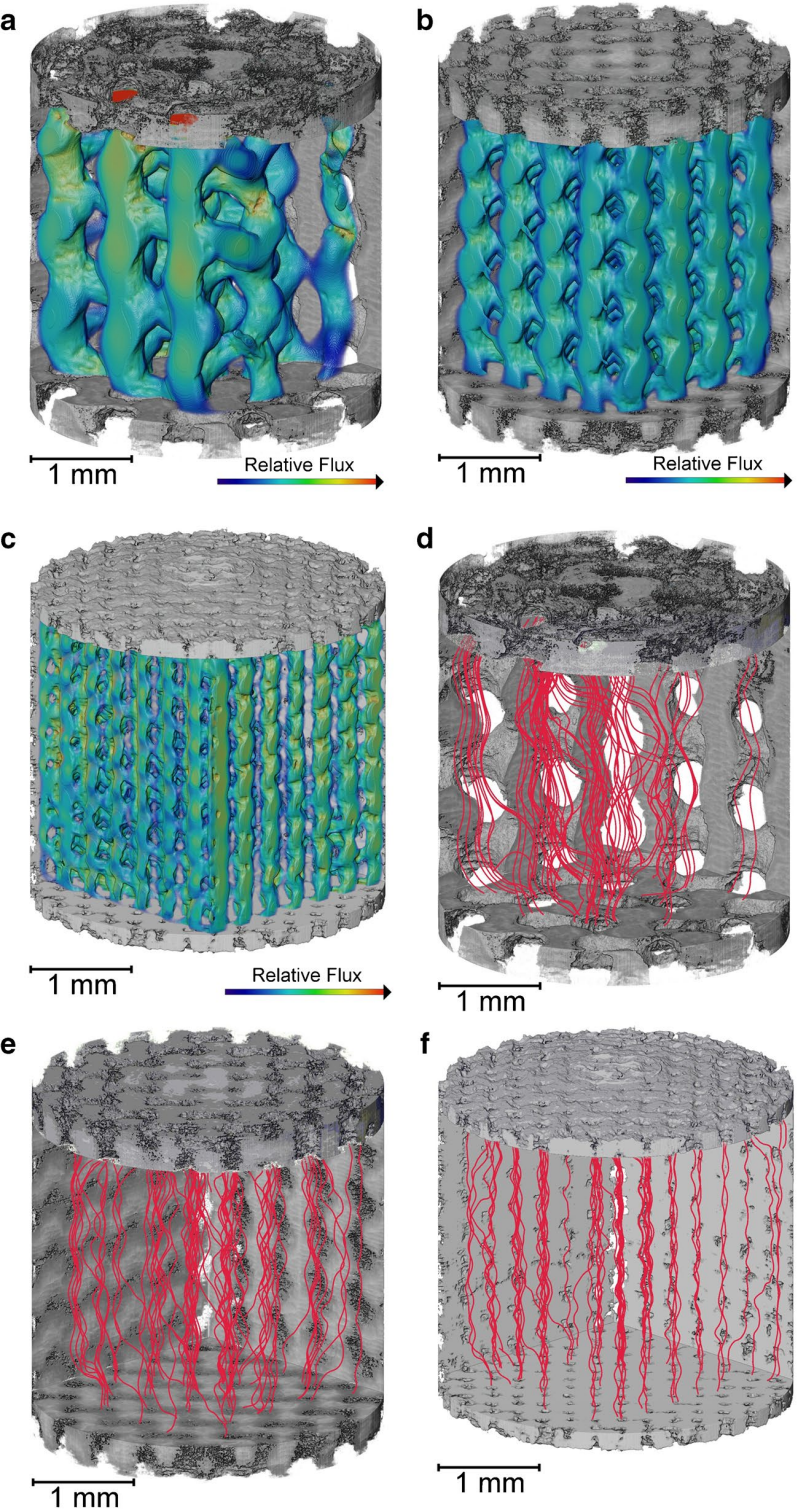
Permeable flow simulation on internal gyroid subvolumes. **a** 500 µm feature size bulk flow analysis from top to bottom. **b** 300 µm feature size bulk flow analysis. **c** 200 µm feature size bulk flow analysis. **d** 500 µm feature size permeable streamlines in red. **e** 300 µm feature size permeable streamlines. **f** 200 µm feature size permeable streamlines

These volumes were segmented into material and void phases to use the porous geometry for permeable flow analysis, enabling simulations to be performed on each geometry as fabricated rather than relying on the idealized design file. Figure 5a–c display bulk permeability analysis, whereby the ordered channels provide consistent flow paths for fluid to traverse through each internal structure. Simulated tortuosity factors of 1.25 to 1.39 were measured from largest to smallest feature size [45]. A greater tortuosity factor corresponds to an increased path length due to the intricate gyroid geometry.

A series of red streamlines where fluid or particles could flow are shown in Fig. 5d–f. The 100 streamlines displayed in each case are representative of the bulk fluid in Fig. 5a–c. In all three cases, the majority of streamlines flow down a single channel as intended, with numerous cases of streamlines traversing to another channel. The interconnecting flow between quasi-vertical channels is most obvious in Fig. 5d through the larger horizontal voids behind the simulated streamlines. Optimizing permeable flow properties is important at this scale to ensure uniform flow whilst maximizing opportunities for species in the fluid to interact with the material phase. The void phase examined at the scale is comparable to the space between chromatography beads at the packed bed scale.

### Nanoporous characterization

**Fig. 6:**
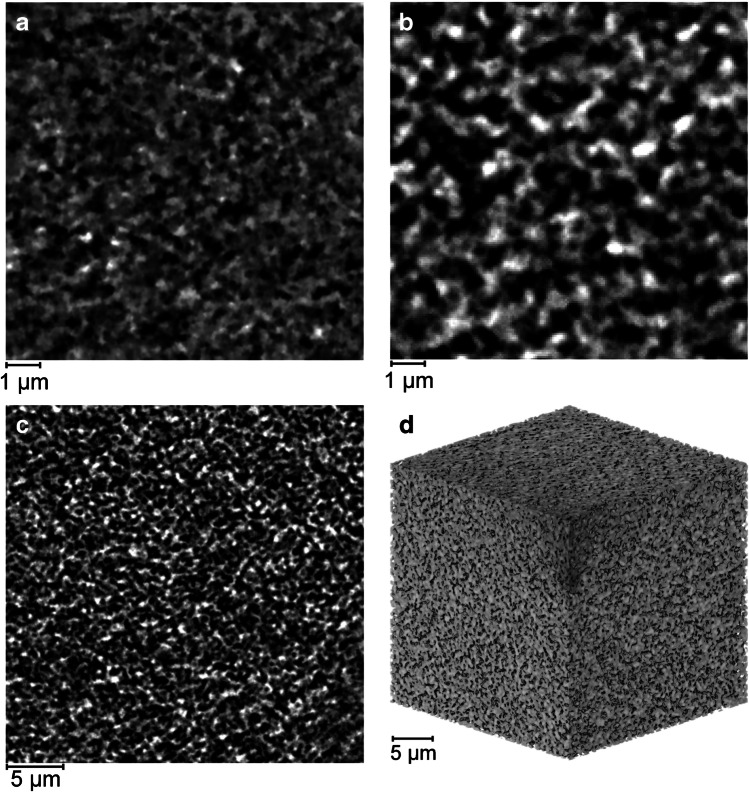
Gyroid nanoscale imaging. **a** 2D slice of a sample imaged at a 16 nm pixel size. 2D slice imaged at a 63 nm pixel size of equivalent size. **b** 2D slice of a sample imaged at a 63 nm pixel size, of equivalent area to Fig. 7a. **c** 2D slice of a sample imaged at a 63 nm pixel size **d** 3D render of a sample imaged at a 63 nm pixel size

Bioseparation media typically have nominal pore ratings in the range of tens to hundreds of nanometers rather than the 500 to 200 µm designed channels as examined here [28]. Each gyroid material phase has an internal porosity that provides the required surface area for product or impurity binding in the diffusive domain, analogous to internal chromatography bead structure. Imaging a small piece of 3D-printed gyroid at a pixel size of 16 nm using nanoscale X-ray CT enabled fine features to be resolved in Fig. 6a; however, the volume generated was deemed to be insufficient due to a greatly restricted field of view.

Therefore, a pixel size of 63 nm was selected for imaging as a suitable balance of high resolution whilst generating a larger volume for analysis and flow simulation. Figure 6b displays an equivalent slice at the lower resolution that still demonstrates a good signal-to-noise ratio and clear differentiation between the black voidage phase and the gray material. Figure 6c shows a much larger field of view at the same resolution, revealing the intricate and detailed substructure within the material phase that is further demonstrated in Fig. 6d.

**Fig. 7:**
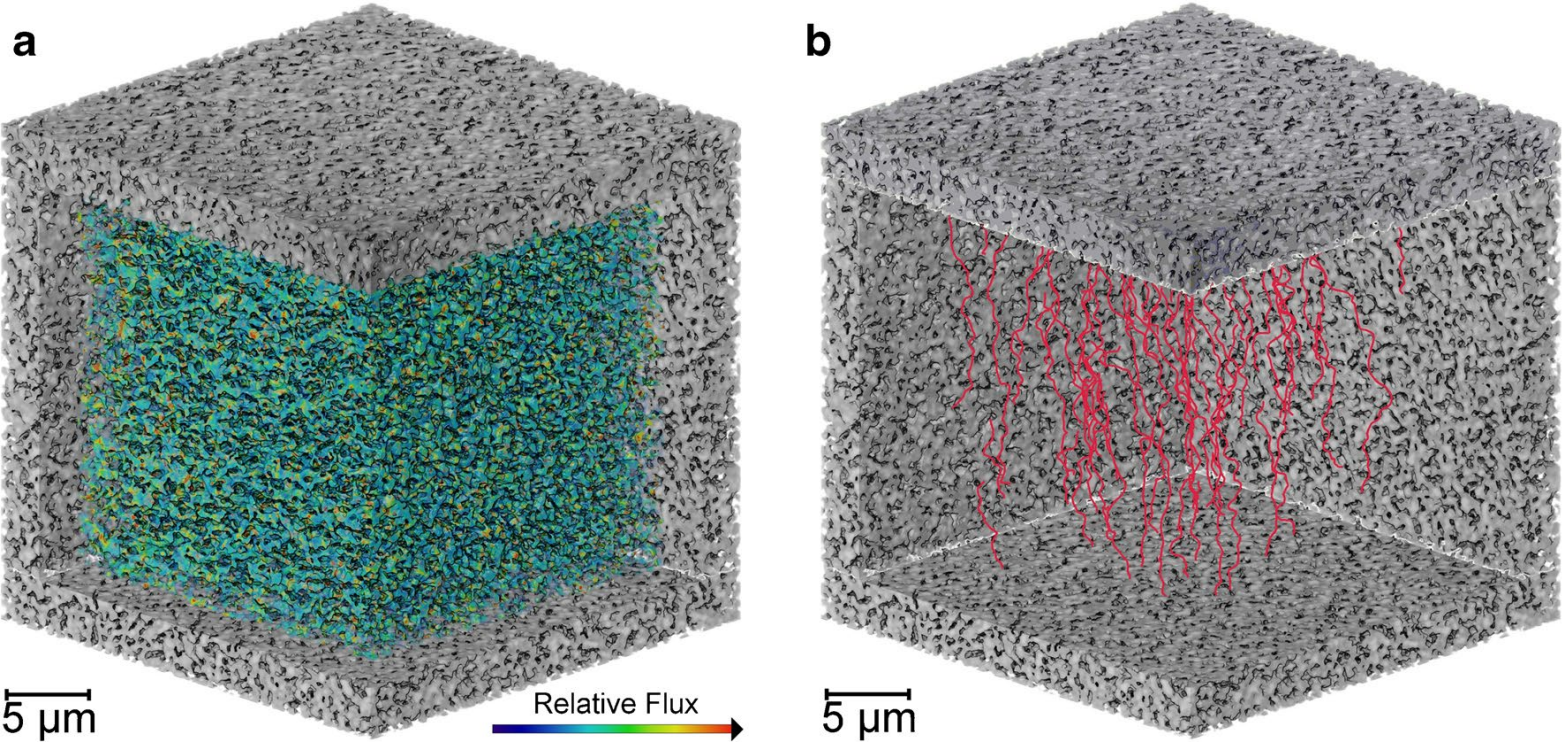
Internal diffusion visualization. **a** Bulk diffusivity flow analysis from top to bottom plane. **b** Diffusive streamlines from top to bottom plane

The tortuosity factor was simulated across all planes within a binarised cube, resulting in an average measurement of 2.52 ± 0.33. Tortuosity is an important factor in governing mass transfer properties; therefore, the internal nanostructure in the material phase provides another length scale that can be optimized for the product and process of interest [13]. By obtaining digital representations of real porous material, more accurate measurements can be made in comparison to conventional techniques. Equation-based derivation using porosity as an input has historically been applied, however does not consider the internal geometry of materials being estimated for tortuosity, and thus for chromatographic quality evaluation, other means are used, e.g., height equivalent theoretical plates [47]. Diffusive flow and streamlines are provided in Fig. 7, suggesting complex yet reasonably consistent geometry without any noticeable areas of bypass. A bulk solution diffusivity of 7.00 × 10^−11^ m^2^s^−1^ resulted in a simulated material diffusivity of 2.17 × 10^−11^ m^2^s^−1^ ± 0.16 × 10^−11^ m^2^s^−1^.

**Fig. 8:**
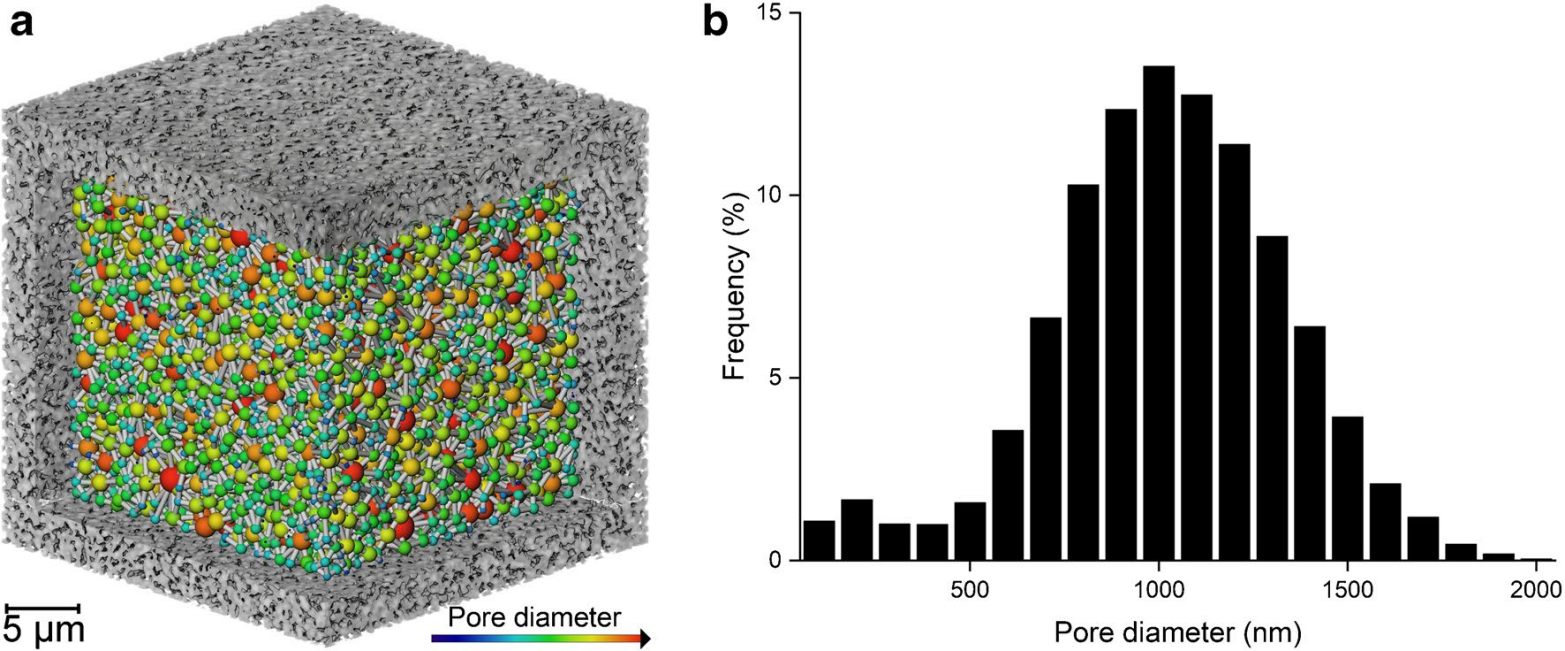
Pore size characterization at a 63 nm pixel size. **a** Pore network mapping. **b** Pore size distribution

The binarised volume enabled pore analysis of the voidage phase. Figure 8a displays a pore network map. Pores can be observed to have a range of sizes and also connect with other pores as represented by sticks between the spheres. The pore size distribution in Fig. 8b measured an average pore diameter of 793 nm ± 315 nm. This pore size distribution suggests larger diameters compared to a previous study that measured an average of 289 nm; however, that approach relied on 2D scanning electron microscopy measurements on the sample surface with a different material composition [13, 16].

By applying a design cycle approach that uses imaging data to inform decisions, pore size characteristics can be specifically tailored to the needs of the product and bioprocess across both permeable and diffusive domains by analyzing real materials. Ensuring that porogens are entirely removed from the material phase through washing steps is necessary to minimize nanostructure variability.

## Conclusion

3D printing has been a transformative fabrication technology for many sectors, however has not yet experienced widespread adoption for bioprocessing purposes in part due to the inherent complexities associated with medicines manufacturing. Whilst this approach still requires development and maturation before manufacturing scale operation can be realized, the ability to produce materials with chemical and physical properties specifically tailored to a product of interest presents several bioprocessing opportunities. The recent emergence of a diverse field of therapeutic modalities such as viral vectors and lipid nanoparticles, which have increased complexities and size when compared to established therapeutic proteins to conventional monoclonal antibodies for bioprocessing, may benefit from novel structures.

Through integrating 3D imaging with 3D printing, structure across multiple length scales can be directly related to function and performance. Structural data from X-ray CT has been found to greatly enhance quantitative information available to inform improvements to the next generation of bioseparation structure prototypes. In future studies, physical properties such as pore size in relation to product morphology will be specifically tailored at both the printed and nanoporous scales to maximize opportunities to create a step change in biopurifications. With the advance of 3D printing technology, and in particular increase in the printing resolution at the micron level, it is forecasted that application of such tailorable 3D printed media will expand also to analytical separations, including use in biochemical profiling.
